# Protocols for Generating Surfaces and Measuring 3D Organelle Morphology Using Amira

**DOI:** 10.3390/cells11010065

**Published:** 2021-12-27

**Authors:** Edgar Garza-Lopez, Zer Vue, Prasanna Katti, Kit Neikirk, Michelle Biete, Jacob Lam, Heather K. Beasley, Andrea G. Marshall, Taylor A. Rodman, Trace A. Christensen, Jeffrey L. Salisbury, Larry Vang, Margaret Mungai, Salma AshShareef, Sandra A. Murray, Jianqiang Shao, Jennifer Streeter, Brian Glancy, Renata O. Pereira, E. Dale Abel, Antentor Hinton

**Affiliations:** 1 Hinton and Garza Lopez Family Consulting Company, Iowa City, IA 52246, USA; egarzalopez@gmail.com; 2 Department of Molecular Physiology and Biophysics, Vanderbilt University, Nashville, TN 37232, USA; zer.vue@vanderbilt.edu (Z.V.); heather.k.beasley@vanderbilt.edu (H.K.B.); andrea.g.marshall@vanderbilt.edu (A.G.M.); tarabia0702@gmail.com (T.A.R.); vanglarry@gmail.com (L.V.); 3 National Heart, Lung, and Blood Institute, National Institutes of Health, Bethesda, MD 20892, USA; prasannakatti.katti@nih.gov (P.K.); brian.glancy@nih.gov (B.G.); 4 Department of Biology, University of Hawaii at Hilo, Hilo, HI 96720, USA; kneikirk@hawaii.edu (K.N.); mbiete@hawaii.edu (M.B.); 5 Department of Internal Medicine, Carver College of Medicine, University of Iowa, Iowa City, IA 52242, USA; jacob-lam@uiowa.edu (J.L.); margaretmungai24@gmail.com (M.M.); salma-ashshareef@uiowa.edu (S.A.); jennifer-streeter-1@uiowa.edu (J.S.); 6 Department of Biochemistry, Cancer Biology, Neuroscience and Pharmacology, School of Graduate Studies and Research, Meharry Medical College, Nashville, TN 37208, USA; 7 Microscopy and Cell Analysis Core Facility, Mayo Clinic, Rochester, MN 55905, USA; christensen.trace@mayo.edu (T.A.C.); salisbury@mayo.edu (J.L.S.); 8 Department of Biochemistry and Molecular Biology, Mayo Clinic, Rochester, MN 55905, USA; 9 Department of Cell Biology, School of Medicine, University of Pittsburgh, Pittsburgh, PA 52013, USA; smurray@pitt.edu; 10 Central Microscopy Research Facility, University of Iowa, Iowa City, IA 52242, USA; jian-shao@uiowa.edu; 11 Fraternal Order of Eagles Diabetes Research Center, Iowa City, IA 52242, USA

**Keywords:** Amira, SBF-SEM, FIB-SEM, segmentation, organelles, 3D reconstruction, 3D imaging, mitochondrial imaging, volume analysis

## Abstract

High-resolution 3D images of organelles are of paramount importance in cellular biology. Although light microscopy and transmission electron microscopy (TEM) have provided the standard for imaging cellular structures, they cannot provide 3D images. However, recent technological advances such as serial block-face scanning electron microscopy (SBF-SEM) and focused ion beam scanning electron microscopy (FIB-SEM) provide the tools to create 3D images for the ultrastructural analysis of organelles. Here, we describe a standardized protocol using the visualization software, Amira, to quantify organelle morphologies in 3D, thereby providing accurate and reproducible measurements of these cellular substructures. We demonstrate applications of SBF-SEM and Amira to quantify mitochondria and endoplasmic reticulum (ER) structures.

## 1. Introduction

Research on the physiology and metabolism of organelles is critical to understanding vital cellular processes such as apoptosis, respiration, and mitosis, and metabolic organelles such as mitochondria and endoplasmic reticulum (ER) have been well characterized. Because these organelles play major roles in regulating homeostasis and ensuring organism survival, it is important to study the relationships between their structure and function. While oxidative phosphorylation in mitochondria is critical for generating ATP, these organelles have functions beyond energetics [1,2,3]. For example, dynamin-related protein-1 (DRP-1) regulates mitochondrial fission and is associated with the early stages of apoptosis [4,5]. Changes in mitochondrial ultrastructures are associated with changes in chemical pathways that regulate calcium, potassium, and other biomolecules [6,7]. Mitochondrial maintenance of calcium homeostasis is also associated with cellular apoptosis; cellular calcium levels affect ER calcium levels, which in turn regulate mitosis [8]. Further, calcium levels play a role in the regulation of the citric acid cycle and calcium signaling that are associated with apoptosis [9,10]. Because mitochondria have a role in apoptosis and control of apoptosis is a component of cancer, a better understanding of mitochondria is critical for cancer treatment [3,8,9,10]. Mitochondria and the ER are essential for cell growth and survival, and they impact many biological functions. Thus, they may serve as targets for pharmaceutical treatments of cancer and neurodegenerative and viral diseases [2,7].

Mitochondria and ER can be imaged by light microscopy and electron microscopy (EM), but despite advances in light microscopy imaging, EM is unparalleled in providing high-resolution images with great ultrastructural detail and 3D visualization [11]. Scanning electron microscopy (SEM) and transmission electron microscopy (TEM) are typically used to study organelle ultrastructure [12,13,14,15]. SEM uses high-resolution backscatter detection to provide detailed information on the surface of a sample. TEM produces nanometer-resolution images by transmitting electrons through an ultrathin section of a sample. Volume electron microscopy, which refers to SEM techniques that can analyze relatively large volume structures, generates near-TEM resolution images using backscatter detection from a block face rather than from an ultrathin section. Three-dimensional visualizations of cellular ultrastructure can be created by serial block-face scanning electron microscopy (SBF-SEM) and focused ion beam scanning electron microscopy (FIB-SEM). With an in-chamber ultramicrotome (for SBF-SEM) or ion beam (for FIB-SEM), samples can be sectioned continuously and the block face imaged through very large volumes in the z-plane. The high-resolution stack of images provides unprecedented resolution for visualization and analysis of ultrastructures in 3D. While FIB-SEM and SBF-SEM are the most common methods, other EM imaging techniques such as automated tape-collecting ultramicrotome scanning electron microscopy (ATUM-SEM) have also been used [16].

Here, we used SBF-SEM, which is a relatively new technique developed at the Max Planck Institute in 2004 by Horstmann et al. that has been used for volume EM for various biological samples [17,18]. In SBF-SEM, automated sectioning of heavily mordanted samples allows for backscatter detection and great depths of sectioning of the block face in the z-plane [19,20]. SBF-SEM generates thousands of individual images providing higher resolution and detection of specific changes in organelle morphology compared to other methods [19,20].

New imaging technologies have allowed high-resolution 3D reconstructions of organelles and tissues. However, in many cases, 2D TEM images are better than SEM. TEM has a resolution of less than 50 pm while SEM image resolution is 0.5 nm, and TEM supports magnification that is nearly 20-fold higher than SEM [12]. Point counting, which is used for 2D micrographs, is a powerful method for obtaining volume [21,22]; however, this method provides only estimates of structures versus the more accurate representation of subcellular structures provided by 3D reconstruction. This is especially true for ultrastructure such as nanotunnels [23]. Three-dimensional reconstruction is especially important for measuring the folds of the inner-mitochondrial membrane known as cristae [7]. Although these folds are typically represented as ribbon-like in a 2D plane, cristae are actually tubular structures that vary in both area and volume [7,24,25]. Three-dimensional reconstructions of mitochondria have provided accurate representations of calcium stores, spatial distributions of metabolites, and the size of cristae [26,27]. Three-dimensional visualizations can be valuable for accurate imaging of ER and mitochondria, other subcellular organelles, and multicellular bodies, including blood vessels and organs [26,28,29,30,31].

High-quality images obtained by SBF-SEM or other data acquisition methods can be reconstructed in 3D using Amira. Amira is a user-friendly application that allows 2D-image slices, known as orthos, to be digitally analyzed, segmented, color-coded, and rendered for 3D reconstruction. Programs for 3D reconstruction of 2D images include ImageJ, Microscopy Image Browser (MIB), Arivis, Imaris, Dragonfly, and Ilastik. Amira has the unique ability to combine segmentation and 3D reconstruction tools to expedite workflow. Amira is highly customizable through an animation creation tool, the ability to assign colors to various organelles, interpolation, compatibility with a wide range of import and export files, and simple workflows with either manual or semi-automated segmentation [32,33] (Supplemental Figure S1). Further modification is possible through scripting interfaces using MATLAB and Python [29], which is important because deep-learning algorithms on Python can expedite the segmentation workflow [34]. The flexibility of Amira provides advantages for both beginners and experienced researchers over less costly open-source software, such as ImageJ. However, Amira has specific hardware requirements and several potential drawbacks (see Limitations; Supplementary Table S1).

We describe the use of Amira for segmentation and 3D reconstruction of images acquired through SBF-SEM that can increase our understanding of structure–function relationships in the context of (1) mutations that alter cellular components, and (2) the therapeutic target potential of cellular components. Here, we present methods for segmenting and measuring mitochondria and ER volume, and for identifying the contact sites between these organelles after knockdown of optic atrophy-1 (OPA1) and mitofusin-2 (MFN-2) versus the controls. Although our paper outlines how Amira can be used specifically for 3D reconstruction of mitochondria in SBF-SEM, Amira can potentially be used to 3D reconstruct any volumetric raw data from any equipment.

## 2. Protocol

This protocol is based on the Microsoft Windows 10 operating system and may differ for other platforms; however, it has been successful using MacOS. We recommend using the current versions of Amira (6.7) and Windows 10.

### 2.1. Installing and Preparing Amira

1. Purchase and download Amira software from ThermoScientific: https://www.thermofisher.com/us/en/home/industrial/electron-microscopy/electron-microscopy-instruments-workflow-solutions/3d-visualization-analysis-software/amira-life-sciences-biomedical.html (accessed on 13 June 2021).
2. Open Amira software.
3. Select **Project View > Open Data** and import the image files to be analyzed from a specific folder.
4. Select **Read Complete Volume into Memory** to ensure all images have been transferred.

**NOTE**: We recommend that ~300 slices, known as orthos, be captured and uploaded; however, not all the images must be segmented for the 3D reconstruction. Although only 50 orthos are typically used, it is important to import excess orthos because structures will not be visible in all orthos, and the best quality slices can be chosen. The user is responsible for choosing these orthos for best results. (Supplementary Table S2, Supplementary Figure S1).

5. Under **Image Read Parameters**, specify **Voxel Size** (nm) of the images according to the size of the files.

### 2.2. Three-Dimensional Segmentation in Amira

1. Under the **Project** subsection, select an ortho slice to analyze.
2. Under the **Segmentation** subsection, select the **Brush** tool and brush size appropriate for the analysis; brush sizes 2 and 3 are commonly used.

**NOTE**: The **Magic Marker** tool (under Segmentation tools) allows automatic segmentation of structures, which can facilitate the workflow. The specificity can be adjusted in the **Properties** tab on the bottom left and should be adjusted to select only the specific structures to be measured. When moving the mouse with the Magic Marker tool selected, pressing ‘shift’ or ‘control’ will add or delete, respectively, additional areas. The **Apply to All Stacks** checkbox allows the Magic Marker for a single ortho slice to be extended to all the slices. Although this method is faster, it is less precise and results in more noise. (Supplementary Table S3; Supplementary Figures S1 and S2).

3. Activate the Wacom tablet device.

**NOTE**: Alternatively, if using the Amira **Brush** tool for **Segmentation**, proceed to step 2.5 using the mouse cursor rather than the Wacom stylus for tracing. We recommended using the **Auto Trace** tool (under **Segmentation** tools) if using a mouse. **Auto Trace** assists in correcting tracing mistakes made when performing segmentation with a mouse. Although a mouse is a suitable alternative, a Wacom tablet with a stylus offers much greater precision while providing great ease in segmentation.

4. Calibrate the Wacom Pen
5. To calibrate, open the **Wacom Tablet Properties** application on the Wacom tablet.
6. Select the name of the stylus being used and then select **Calibrate**.
7. In each corner of the Wacom screen, a cross will appear. Using the stylus, select the middle of the cross.

**CAUTION**: Calibrating the Wacom pen is important; otherwise, the stylus may be inaccurate and significant errors in segmentation may result. We recommend recalibration every 30–60 min; the pen’s accuracy degenerates over time resulting in a discrepancy between the location of the stylus tip and the selection on the screen.

8. Drag the Amira software window until it is visible onto the Wacom screen.
9. Using a stylus and the Wacom tablet, outline all instances of one type of structure (e.g., mitochondria or blood vessels) in the ortho slice.

**CAUTION**: The segmentation borders of separate structures must not touch because Amira will automatically merge these structures into one. To prevent the merging of close structures, the brush size can be adjusted to 1 to decrease the width of the brush tool. Alternatively, the **shrink** option from the **display controls** can be used to reduce the threshold that Amira uses to combine structures.

10. When all structures of one type have been segmented, press **F** to segment the areas of the outlined structures.
11. Select a material color for the organelle type.

**CAUTION**: Colors must be used consistently for all organelles and must follow the same pattern because the colors of separate materials cannot be changed at the end. All instances of one type of organelle should be segmented at the same time in a consistent color; afterward, the color can be changed. This avoids the joining of different organelles that leads to inconsistent 3D reconstructions in the final rendered image. If one desires to pseudocolor individual organelles, one should only segment one organelle type at a time and different colors should be used for each individual organelle.

12. To change the shading of an area for better visibility, press **D** to change the settings between full color, outline, or gradient.

**NOTE**: Using the **D** button to visually change the pattern of an area, especially if a lighter color is being used, helps to prevent the accidental segmentation of a structure multiple times which may lead to inaccurate results.

13. Under **Segmentation > Selection**, select the **+** icon to add the selection.
14. Repeat this process for each unique structure type on the ortho slice.
15. Repeat this process for each ortho slice ensuring that all structure types consistently use the same material color.
16. Using the **Materials** menu, colors can be adjusted, locked, or made invisible by clicking on the color panel, the 2D or 3D checkmarks, and/or the lock button.
17. Segmentation.
18. This segmentation protocol is based on the type of organelle; however, it can be based on individual organelles (i.e., segmentation of each mitochondrion separately).
19. To segment organelles individually, repeat Step 2.4 to create a new material for each individual organelle versus each type of structure.

**CAUTION**: It is recommended to perform individual segmentation of one organelle type at a time to avoid confusion that may result from working with the different material colors.

### 2.3. Three-Dimensional Reconstruction of Segmented Structures in Amira

1. Go to the Project Menu.
2. Click on **Selection Labels > Generate Surface**.
3. Select **Apply**.
4. Rename the newly generated selection cell with the “**. surf**” suffix and click **Surface View**.
5. The 3D structures should appear over an ortho slice.
6. Disable the overlaid ortho image. Toggle off to hide the image by clicking on the orange rectangle labeled **Ortho Slice** and selecting the **toggle button** (blue square) under the **Properties** menu.

**NOTE**: Now adjust the segmented objects that are shown and their colors. While surface view is selected, under **Properties > Materials**, specific Materials can be selected. To remove materials, select **Properties > Buffer > Remove** for structures that are no longer needed. Alternatively, the visibility of materials may be toggled on and off by clicking the blue boxes next to the materials. (Supplementary Table S4, Supplementary Figure S1).

7. Make scale bars.
8. Right-click in the gray area under the **Project** subsection and select the option **Scalebars**.
9. Leave only the frame x-axis active.
10. Set the line width and font to something easy to read; 4 px and Arial 18 pt. font is recommended.
11. Set the position and size to the bottom right corner; the recommended scale is 500 nm.

### 2.4. Creating a Video/Animation in Amira

1. Right-click in the gray area under the **Project** subsection and select **Camera Orbit**.
2. Click on the newly created Camera Orbit box and select **Movie Maker**.
3. With Camera Orbit selected, select a parameter under **Action**, such as the **Up Direction** to create a simple animation.
4. Alternatively, right-click in the gray area under the **Project** subsection and select **Camera Path**.
**5**. **Camera Path > Camera Path Editor** will pop up a new viewer.
**6**. **Camera Path > Add** will add new keyframes in the viewer. The positions of the keyframes can be altered in the new viewer by moving the cuboidal objects. Add as many keyframes as desired and use the cursor to adjust the path between the keyframes.
7. Adjust the time between the keyframes in the **Camera Path** menu. This will allow more complex and specific animation movements.
8. With the **Movie Maker** label selected, choose the movie parameters, such as File Format (MPEG) and Size (1080P optional).
9. Click **Apply** at the bottom left.
10. **Save** video. See (Supplementary Table S5, Supplementary Figure S1)

### 2.5. Quantification

1. Three-dimensional length and other length measurements can be set by selecting the ruler icon under the tool icon when viewing the **Project** view.
2. Ensure a scale has been set in Amira.
3. Select the **Line** measurement tool and click on the desired surface to measure.
4. Click on the two points to be measured. For 3D length, this should be the Feret’s diameter.
5. The length will be calculated automatically. Units may be changed by clicking on the number that appears on the line measurement.
6. For other measurements, right-click in the gray area under the **Project** subsection and select **Label Analysis**.
7. Click on the newly created Label Analysis box and ensure that the properties are visible at the bottom left of the screen.
8. Under the **Data** and **Intensity Image** sections, ensure that the desired data are chosen for analysis, and ensure for all 3D measurements that **3D** is selected under the **Interpretation** section.
9. Under the **Measures** section, click on the “**...**” to choose quantifications.
10. In the newly opened Measures window, click the ruler icon next to **User Measures**, and name the new group of measurements.
11. Add all relevant measurements to the new measurement group.
12. Area3d, Volume3d, and perimeter measurements, used for quantification in this study, are default measurements in Amira. Select them from the **Native Measurements** box and click the ruler icon in the middle to add to the measurement group.
13. Sphericity is a custom measurement that can be added in the **Measure Editor** menu that opens when creating a new measurement group. In the output menu, type in the equation for sphericity according to Amira convention: (π**(1/3) *(6*Volume3d) **(2/3)/Area3d.
14. Other quantifications are available as default measurements or can be added using an equation via the method presented in step 5.7.2.
15. Once all measurements are added, click the **Ok** button to save this new group for present and future analysis.
16. In the bottom left, select the newly created measurement group under the **Measures** section.
17. Click the **Apply** button at the bottom left of the properties screen. Data can then be copied to create graphs in other software, such as GraphPad. See (Supplementary Table S6, Supplementary Figure S1).

## 3. Results

This protocol provides the steps for 3D reconstruction and animation of organelles, subcellular structures, and organ structures. We used this protocol to perform 3D reconstructions of mitochondria and ER.

### 3.1. SBF-SEM Reveals Mitochondrial Changes in OPA1 smKO-Derived Skeletal Muscle

**Figure 1:**
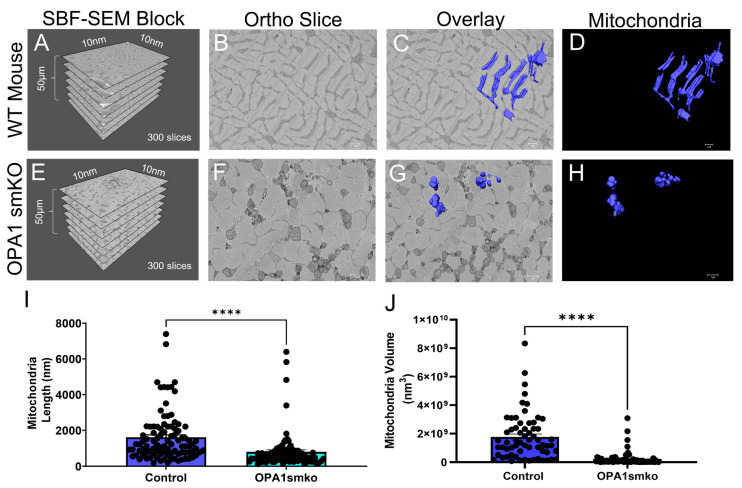
Skeletal muscle specific knockout of *OPA1* (*OPA1* smKO) in mouse leads to changes in mitochondrial morphology in the mouse. The 3D distribution of single continuous and stationary mitochondria (blue), reconstructed from serial block facing-scanning electron microscopy (SBF-SEM) image stacks of gastrocnemius muscle from *OPA1* smKO mouse (**A**–**H**). (**A**) The dimensions of the captured tissue in wild type mouse and (**E**) OPA1 smKO, (**B**,**F**) along with an example ortho slice for each. (**C**) The overlay of the 3D surface rendering of mitochondria in a wild type mouse, on top of a representative ortho slice and (**D**) the 3D surface rendering of mitochondria alone. (**G**) The overlay of the 3D rendering of mitochondria in OPA1 smKO, on top of a representative ortho slice and (**H**) the 3D surface rendering of mitochondria alone. (**I**,**J**) The 3D mitochondrial length and volume decreased (**** *p* < 0.001) upon OPA1 smKO.

OPA1 is an inner-mitochondrial membrane that regulates mitochondrial dynamics by promoting fusion [4,5,35,36,37]. While mitochondrial fission divides mitochondria reducing their volume, mitochondrial fusion increases their volume. OPA1 not only promotes fusion but maintains tight crista junctions and regulates apoptosis [4,35,36]. Three-dimensional reconstruction reveals the structural dynamics of mitochondrial fusion and fission events that are lost in the 2D plane [4,5]. We expected to observe structural changes in mitochondria, such as smaller size and reduced connectivity after knocking out OPA1. High-quality 3D reconstructions of gastrocnemius muscle from *OPA1* smKO mouse using SBF-SEM (Figure 1A–H, Supplementary Videos S1–S2) were used to quantify and measure the mitochondria. Tissue dimensions (Figure 1A,E) and a representative ortho slice image (Figure 1B,F) are shown. Three-dimensional reconstruction data are presented in two ways. Ortho slices overlaid with a 3D reconstruction provides a way to visualized representative images alongside their reconstructed organelles (Figure 1C,G), while the isolated 3D reconstruction images provide finer details (Figure 1D,H). More details are provided in the videos that show the mitochondria from several angles (Supplementary Videos S1–S2). Both 3D mitochondrial length and volume were substantially decreased with OPA1 ablation (Figure 1I,J) in OPA1 smKO skeletal muscle compared to the wild-type control. These 3D data indicate that OPA1 mutants produced smaller, fragmented mitochondria due to increased fission from reduced OPA1 levels.

### 3.2. Three-Dimensional Reconstruction Allows for Identification of Mitochondria-ER Contact Sites (MERCs)

The mitochondria and the ER play critical roles in cellular processes such as apoptosis, metabolism, and progression through the cell cycle [1,2,3,38], and there is much interest in the specific locations of the MERCs. MERCs are important because they mediate the transfer of calcium from the ER to the mitochondria and serve as biosignaling sites, among other important functions [39,40,41,42]. Calcium and lipid signaling mediated by MERCs are also important for mitochondrial fission and fusion. Additionally, 3D reconstructions of MERCs may provide a better understanding of changes in ER-mitochondrial communication that result from disease.

**Figure 2:**
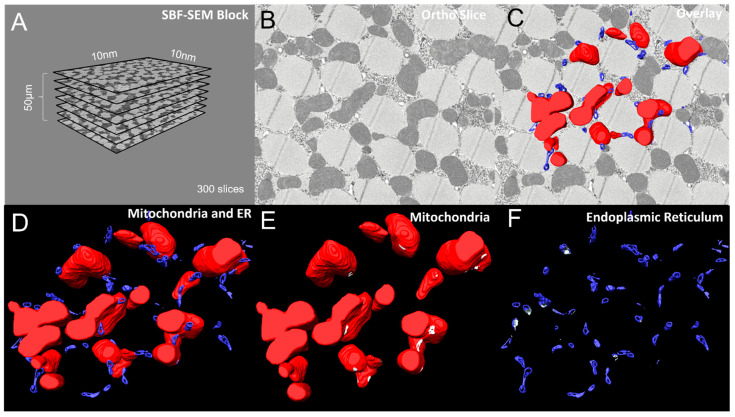
6-panel presentation of 3D reconstruction images and ortho slices from wildtype *Drosophila* flight muscle. This figure is an example of how to present the ortho slices and the 3D reconstruction images. This example shows 3D reconstruction of several organelles in *Drosophila* flight muscle. (**A**) On the left, several representative ortho slices are presented. The dimensions and amounts of ortho slices for data acquisition and conversion to 3D models are shown. (**B**) The raw image of an ortho slice. (**C**–**F**) Mitochondria are colored red, ER are colored blue, and MERCs are colored white. These data are best presented in several ways. (**C**) 3D reconstruction overlaid over the ortho image allows for better visualization of the specific structures in the ortho image that are reconstructed. (**D**) In contrast, the 3D reconstruction not overlaid on the ortho image allows for better visualization of interactions between the 3D structures. (**E**,**F**) Finally, Amira also allows for the graying out of specific structures such that only mitochondria or ER are shown in the 3D reconstruction. This is useful to view otherwise difficult to see areas including MERCs.

Here, we provide 3D reconstructions of MERCs in *Drosophila* flight muscle including standard 3D reconstruction data with an analysis of the dimensions of the ortho stacks (Figure 2A) and a representative ortho slice (Figure 2B) overlaid with MERCs (Figure 2C). These panels include descriptions of data acquisition and sampling of the base images used for segmentation. The 3D reconstruction showing the mitochondria and ER provides information on the volumetric spaces of the contacts between mitochondria (red) and ER (blue) (Figure 2D).

Alternatively, we provide an animation generated in Amira (Supplementary Video S3). Both representations show many MERCs with complete visualization of both the mitochondria and the ER. These two representations of qualitative data improve the analysis and interpretation of the effects of specific genes on MERC formation, structure, and maintenance. While the MERCs in the 3D reconstruction are labeled in white (Figure 2E,F), the data provided as a video animation provide a less cluttered 3D view that shows MERCs clearly. Furthermore, 3D animation provides more detailed illustrations of the sizes of MERCs throughout the depths of the mitochondria (Figure 2E,F, Supplementary Video S3).

### 3.3. Three-Dimensional Reconstruction Shows Organelle Morphology Changes upon Knockdown of MFN-2

Mitofusin-2 (MFN-2) is a GTPase needed for mitochondrial fusion and serves as a physical tether between the ER and mitochondria, thereby mediating their connections and the calcium exchange between them [39,40,43,44,45]. To confirm the utility of our method for measuring changes in ER, mitochondria, and MERCs, we knocked down MFN-2 in myotubes. Thus, loss of MFN-2 causes mitochondrial dysfunction and ER stress [46,47]. Although the role of MFN-2 in MERCs is controversial, many studies have shown that the loss of MFN-2 decreases MERC distances [43,44,48,49,50]. Because MERCs are central to cellular homeostasis, MFN-2 is important for the effective functioning of cells. Dysfunctional MERCs have been linked to cancers and metabolic syndromes; thus, it is critical to find methods to measure them [48]. We developed 3D-reconstruction methods to measure MERCs and explored the debated role of MFN-2 on MERCs.

**Figure 3:**
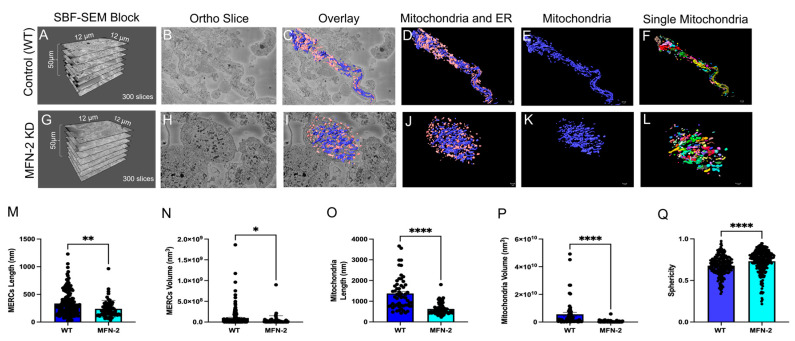
MFN-2 knockdown in myotubes results in smaller mitochondria and ER, with shorter MERCs. MFN-2 deficiency in myotubes leads to changes in mitochondrial (blue) and ER (pink) morphology in the mice. (**A**–**D**) The dimensions of SBF-SEM tissues, isolated ortho slice, 3D reconstruction overlay, and isolated 3D reconstruction in wild type myotubes. (**E**) Wild type myotubes 3D reconstructions are also shown with mitochondria individually colored and (**F**) ER individually colored. (**G**–**J**) The dimensions of SBF-SEM myotubes, isolated ortho slice, 3D reconstruction overlay, and isolated 3D reconstruction in *MFN-2* deficient-myotubes. (**K**) 3D reconstructions of mitochondria in a single color and (**L**) individually colored. (**M**) When measuring MERCs, MERC length and (**N**) MERC volume both decreased. Furthermore, the (**O**) mitochondria length, (**P**) mitochondrial average volume also decreased. (**Q**) The sphericity of mitochondria additionally decreased. Significant differences are indicated by asterisks; *, **, and **** indicate 405 *p* ≤ 0.05, *p* ≤ 0.01, and *p* ≤ 0.0001, respectively.

High-quality 3D reconstructions of MFN-2 deficient myotubes using SBF-SEM were used to quantify and measure the mitochondria. We provide images showing the dimensions of the cut tissues and an ortho slice, along with the 3D reconstruction overlay, and several 3D reconstructions (Figure 3A–L). While using a single color per organelle can be effective (Figure 3D,E,J,K), viewing changes in individual mitochondria in the MFN-2 KD through pseudo coloring (Figure 3F,L) provided additional ways to view and measure mitochondria and MERCs. By quantifying the changes in the MFN-2 deficient myotubes (Figure 3M–P), we determined that lengths and volume decreased as mitochondria became smaller and less connected compared to the wild type, as did mitochondrial sphericity (Figure 3Q). This suggests that abnormalities in fusion result in dysfunctional mitochondria. The decreases in MERC length and volume (Figure 3M,N) are indicative of changes in MERCs which have been shown in previous studies [43,44,48,49,50].

## 4. Discussion

Research on cardiovascular health and diabetes demonstrates that ultrastructural imaging is essential to understanding how genes, proteins, and other macromolecules alter organelle morphology [51,52,53], and several methods have been used to quantify the morphology of organellar structures [52,53,54]. Here we provide a methodology for 3D reconstruction using SBF-SEM, which is common in biomedical research, and a protocol for using Amira software that may be printed for lab use and reference (Supplemental Material S1). This protocol provides qualitative and quantitative metrics for sample analysis. The findings presented here demonstrate the power of combining SBF-SEM and Amira software; however, further research and standardization of other 3D reconstruction methods are necessary.

Three-dimensional models based on 3D reconstruction provide greater detail in the 3D plane compared to 2D microscopy. Further, animations provide greater detail for the 3D models than do traditional figures (Supplementary Videos S1–S5). Our 3D reconstruction animations reveal the detail and complexity of the models that Amira can produce when keyframes and camera path options are used effectively (Videos S1–S2). For example, for the 3D reconstruction of MERCs, the camera panned across all organelles from prescribed angles to identify all potential MERCs, which would go unseen in traditional images (Supplementary Video S3; Figure 2D–F). Key changes in the mitochondria in cells with OPA1 smKO or MFN-2 KD were clearly visible in videos (Figure 1 and Figure 3, Supplementary Videos S1–S2, S4–S5).

Qualitatively, MERCs that specify the location where lipid and calcium homeostasis primarily occurs were observed clearly in the animations and the 3D reconstructions of mitochondria and ER (Figure 2E,F, Supplementary Video S3). These 3D reconstructions allowed for statistical analysis of metrics that cannot be obtained as reliably in 2D images [21,22] (Figure 3M–Q) under various conditions such as in cells from strains with gene knockdowns. This opens avenues for future research in which cells under various conditions can be analyzed through EM with 3D analyses using SBF-SEM and Amira software [24,25,55].

Other techniques for image acquisition may be used with or as an alternative to SBF-SEM. In the past, serial section TEM (ssTEM) with its simpler equipment requirements was the technique of choice [52]. However, the heavy metals on the surfaces of structures limit the resolution in ssTEM, and, lacking automation, it is not suitable for large numbers of samples because manual processing reduces reliability and reproducibility [52,53]. The resolution of SBF-SEM is less than 10 nm in the x-axis and y-axis, while the resolution ssTEM is less than 1 nm; however, in the z-axis, the resolution is greater for SBF-SEM at less than 50 nm compared to around 60 nm for ssTEM [15,54]. Thus, SEM may provide better 3D reconstructions.

FIB-SEM and SBF-SEM both allow automation of imaging; however, FIB-SEM, which uses an ion beam for cutting, provides greater resolution along the z-axis compared to SBF-SEM, which uses a diamond knife for cutting [52]. Thus, FIB-SEM provides finer structural details for cristae and nanotunnels than SBF-SEM. While both methods provide high resolution they also destroy the sample being analyzed [52]. ATUM-SEM, typically used in neuroscience, is a powerful, automated EM technique in which serial sections collected on a carbon-coated tape are imaged by SEM [16,23,53]. While most ssTEM techniques require skill and are labor-intensive, FIB-SEM, SBF-SEM, and ATUM-SEM are automatized and provide high-resolution 3D images [16,52,53,54] dramatically improving the interpretation of the morphology of tissue and cell samples. Amira software and other similar software (Supplementary Table S7) can be used for FIB-SEM, SBF-SEM, and ATUM-SEM images [23,56,57,58]. For studies with large samples, SBF-SEM is faster and offers larger fields of view than FIB-SEM [54]: thus, we combined SBF-SEM with the versatility, ease of use, and features of the Amira software for analysis of the SBF-SEM images. Detailed comparison between many of the most common methods for data collection, all of which may be employed for data collection for Amira, are found in Supplementary Table S8.

Amira offers a variety of useful features designed for EM data including segmentation, visualization, and quantification of 3D and 4D imaging data [33]. Amira imports microscopy-specific formatted files, which makes for seamless processing [33], and, unlike some competitors, Amira imports TIFF, JPG, PDF, and DICOM files [32]. With a powerful computer, Amira processes files quickly, reconstructing entire organisms in less than thirty minutes [28,59]. Amira allows researchers to create 3D PDF files of objects using image alignment and color adjustment techniques [59,60]. Amira provides interactive high-quality volume visualization with orthogonal and oblique slices, volume and surface rendering, and isolines and isosurfaces for more advanced customization [28]. Following segmentation, Amira provides post-image processing and analysis, including colocalization, photobleaching correction, and 3D visualization. While Amira is user-friendly, it allows advanced users to control the statistical analyses by customizing protocols through MATLAB scripts and by outputting data to Excel (Figure 1, Figure 2 and Figure 3 show statistical analyses performed using Amira).

In general, Amira has many quality improvements that reduce the user workload. Amira offers the ability to reduce noise and artifacts in imported images and allows direct manipulation of 3D images for export [61]. It offers 2D and 3D image filtering to allow segmented ultrastructures to be included in the images while the background is removed (See Videos S1–S5 and Figure 1, Figure 2 and Figure 3 that demonstrate the removal of the background using Amira). For larger data sets such as a time series, Amira can automate the selection of motion models. It provides customized workflows that can be set to detect and render objects such as filaments and microtubules, reducing the time required to analyze data [61]. Amira provides a novel hybrid file format that reduces the computer storage space required to perform reconstructions while retaining the original files and also protects from loss of work if the software or system crashes [61]. Amira provides useful features throughout the workflow from image segmentation to complete animation of the 3D reconstructed images [32] (See Figure 1 for panel types that can be used in Amira). Amira 3D structures can be created easily and can then be used for more complex customized animations that provide information on structural dynamics [33] (See Videos S1–S5 and Figure 1, Figure 2 and Figure 3 that show 2D images and 3D reconstruction animations created with Amira). Thus, Amira provides powerful software tools for the analysis of SBF-SEM images to create high-quality 3D reconstructions of organelles.

## 5. Limitations

Although 3D reconstruction in combination with Amira is a powerful tool, there are some limitations [28,29,30]. First, both Amira and the computer, which needs a powerful processor and graphics card, are expensive (Table S6). Other 3D reconstruction software may be more ideal for certain users (see Table S8 for a comparison of the most commonly used software). Many offer the same options for export, have the ability to interface with other software, and have different layouts and workflows, so a large part of the decision may come down to user preface. Second, manual segmentation can be slow; tracing structures by hand produces very accurate results, but it can take tens or hundreds of hours depending on the structure of interest, their number, and the size of the dataset [33]. However, skilled users may be able to automate segmentation. Third, because the samples are fixed, this method provides a single snapshot of subcellular structures and cannot follow changes in organelle morphology over time. Therefore, other methods, such as organelle staining and live imaging, may be required. Because the sample is destroyed as it is segmented, SBF-SEM cannot be repeated on the same sample, and the sample cannot be reused for further image acquisition. Despite these limitations, the method described here provides comprehensive 3D quantification of organelle morphology.

### Online Methods

A listing of reagents/resources with their unique identifier and where to find them.

**Table:** 

Reagent or Resource	Source	Identifier
Chemicals, peptides, and recombinant proteins		
2% glutaraldehyde in 0.1 M cacodylate buffer	N/A	N/A
3% potassium ferrocyanide	Sigma	Cat# P3289
0.1% thiocarbohydrazide	Electron Microscopy Sciences	Cat# 21900
2% osmium tetroxide	Electron Microscopy Sciences	Cat# 19112
1% uranyl acetate	Electron Microscopy Sciences	Cat# 22400-2
0.6% lead aspartate solution	MP Biomedicals	Cat# 155180
HPLC grade acetone	N/A	N/A
Epoxy 812 hard resin	Electron Microscopy Sciences	Cat# 14900
Experimental Models: Organisms/strains		
Mouse: C57B16	Mayo Clinic	N/A
*Drosophila*: *Mef2 Gal4*	VDRC (Vienna) *Drosophila* stock center and Bloomington *Drosophila* stock center.	BS# 27390
*Drosophila*: *W^1118^*	VDRC (Vienna) *Drosophila* stock center and Bloomington *Drosophila* stock center.	N/A
*Drosophila*: *Opa-1-like*	VDRC (Vienna) *Drosophila* stock center and Bloomington *Drosophila* stock center.	N/A
Software and Algorithms		
Amira Software	Thermo Scientific/Amira [61]	RRID: SCR_007353
Image J	Schneider et al. (2012) [62]	https://imagej.nih.gov/ij/ (assessed on 3 June 2021)
Ilastik	Berg et al. (2019) [63]	https://www.ilastik.org/publications.html (assessed on 3 June 2021)

## 6. Resource Availability

### 6.1. Lead Contact

Further information and requests for resources or reagents should be directed to and will be fulfilled by the lead contact, Dr. Antentor Hinton Jr. (antentor.o.hinton.jr@vanderbilt.Edu).

### 6.2. Materials Availability

This study did not generate any new, unique reagents.

### 6.3. Data and Code Availability

Any additional information required to reanalyze the data reported in this paper is available from the lead contact upon request.

## 7. Experimental Model and Subject Details

### 7.1. Animal Care and Tissue Isolation

A total of 8 male C57Bl6 mice at 12 weeks of age were used to analyze the impact of OPA1 knockout on mitochondrial structure and networking (*n* = 3–5 per group). Mice were under a 12:12 h light:dark cycle with ad libitum access to standard chow and water. All procedures were performed using humane and ethical protocols approved by the University of Iowa Institutional Animal Care and Use Committee following the National Institute of Health Guide for the Care and Use of Laboratory Animals. Mice were anesthetized using a mixture of 5% isoflurane/oxygen and the gastrocnemius muscle was excised and cut into 1 mm^3^ pieces before proceeding to the SBF-SEM protocol.

### 7.2. Fly Strains and Genetics

Genetic crosses were performed on yeast corn medium at 24 °C unless otherwise stated. *Drosophila* strain *W^1118^* was used as the control. *Mef2-Gal4* (III) was used to drive the muscle-specific *Opa-11-like* (OPA1) knockdown (KD). *Tub-Gal80^ts^* and *Mef2 Gal4* (BS# 27390) were used for the conditional muscle-specific *Opa-1-like* KD. Genetic crosses were set up at 18 °C and then shifted to 29 °C at the larval stage. Stocks were obtained from the VDRC (Vienna) *Drosophila* stock center and the Bloomington *Drosophila* stock center. All chromosomes and gene symbols are as described in Flybase (http://flybase.org, accessed on 7 July 2021).

### 7.3. Serial Block Facing-Scanning Electron Microscopy (SBF-SEM) Processing of Drosophila and Mouse Muscle Fibers

For mice, C57BL/6J males were anesthetized via the use of 2% isoflurane. The hair and skin were removed, and the hindlimbs were incubated in 2% glutaraldehyde with 100 mM phosphate buffer for 30 min. Following this, the gastrocnemius muscle was cut into 1 mm^3^ cubes and incubated for 1 h in a 2.5% glutaraldehyde, 1% paraformaldehyde, 120 mM sodium cacodylate solution. *Drosophila* tissues were fixed with 2% glutaraldehyde in 0.1 M cacodylate buffer and processed using a heavy metal protocol adapted from a previously published protocol [14,64].

Following preparation, both tissue types were washed three times with 100 mM cacodylate buffer at room temperature and were immersed in 3% potassium ferrocyanide and 2% osmium tetroxide for 1 h at 4 °C, then treated with filtered 0.1% thiocarbohydrazide for 20 min, 2% osmium tetroxide for 30 min, and left overnight in 1% uranyl acetate at 4 °C; three de-ionized H_2_O washes were performed between each step. The next day, samples were immersed in a 0.6% lead aspartate solution for 30 min at 60 °C and dehydrated in graded acetone. Dehydration was performed by sample being incubated in 20%, 50%, 70%, 90%, 95%, and 100% ethanol, in that order, for five minutes each. Tissues were impregnated with epoxy Taab 812 hard resin, embedded in fresh resin, and polymerized at 60 °C for 36–48 h. After polymerization, blocks were sectioned for TEM to identify areas of interest, trimmed to 0.5 mm × 0.5 mm, and glued to aluminum pins. Pins were placed into an FEI/Thermo Scientific Volumescope 2 SEM, a state-of-the-art SBF imaging system. For 3D EM reconstruction, thin (0.09 µm) serial sections, 300–400 per block, were obtained from the blocks that were processed for conventional TEM. Serial sections were collected onto formvar coated slot grids (Pella, Redding CA), stained, and imaged as described above. Segmentation of SBF-SEM reconstructions was performed by manually tracing structural features on sequential slices of micrograph blocks. Images were collected from 30–50 serial sections that were then stacked, aligned, and visualized using Amira to make videos and quantify volumetric structures.

### 7.4. Quantification

Many parameters, including volume and surface area may both automatically be measured by Amira. Sphericity Length is measured through the Feret’s Diameter, which refers to the distance between any two most distant points in a 3D object. Sphericity refers to the adherence of the object to that of a sphere and is calculated by the equation: (π^1/3^ (6*V*)^2/3^)/*SA*, where *V* refers to the volume of the object and *SA* refers to the surface area of the object. Other useful metrics are available in Amira that are not used here which include mitochondrial branching index and is calculated by the following equation: *SA*^3^/16π^2^*V*^2^ [23]. Mitochondrial complexity measures the ratio between transverse and longitude tissue surrounding the mitochondria [23]. Barycenter and perimeter, to name a few, are other viable metrics that may be calculated in Amira.

### 7.5. Data Analysis

Data were presented as the mean of at least three independent experiments with similar outcomes. Results were presented as mean ± standard error with individual data points shown. Data were analyzed using an unpaired *t*-test. If more than two groups were compared, one-way analysis of variance (ANOVA) was performed, and significance was assessed using Fisher’s protected least significance difference test. GraphPad PRISM and Statplus software packages were used for *t*-tests and ANOVA analyses (SAS Institute: Cary, NC, USA). For all statistical analyses, *p* < 0.05 indicated a significant difference. Higher degrees of statistical significance (**, ***, ****) were defined as *p* < 0.01, *p* < 0.001, and *p* < 0.0001, respectively.

## Supplementary Materials

The following are available online at https://www.mdpi.com/article/10.3390/cells11010065/s1, Figure S1: Printable flowchart of the optimized protocol for organelle quantification using Amira, Figure S2: Sample of the Amira user interface. Table S1: Required hardware for 3D reconstruction of organelles and organs using Amira. Table S2–S6: Visualized step-by-step guide for organelle quantification using Amira. Table S7: Major 3D reconstruction software comparison by cost, segmentation options, and other relevant information. Table S8: Basic overview, advantages, and disadvantages of main methods of collecting data for 3D reconstruction. Video S1: Wild-type Mouse Gastrocnemius Muscle Mitochondria. Video S2: OPA1smko Mouse Gastrocnemius Muscle Mitochondria. Video S3: Drosophila Mitochondria and Endoplasmic Reticulum Contacts. Video S4: Wild-type myotube mitochondria Video S5: MFN-2 Deficient Myotube Mitochondria.
