# Correction: Garza-Lopez et al. Protocols for Generating Surfaces and Measuring 3D Organelle Morphology Using Amira. *Cells* 2022, *11*, 65

**DOI:** 10.3390/cells12101356

**Published:** 2023-05-10

**Authors:** Edgar Garza-Lopez, Zer Vue, Prasanna Katti, Kit Neikirk, Michelle Biete, Jacob Lam, Heather K. Beasley, Andrea G. Marshall, Taylor A. Rodman, Trace A. Christensen, Jeffrey L. Salisbury, Larry Vang, Margaret Mungai, Salma AshShareef, Sandra A. Murray, Jianqiang Shao, Jennifer Streeter, Brian Glancy, Renata O. Pereira, E. Dale Abel, Antentor Hinton, Jr.

**Affiliations:** 1 Hinton and Garza Lopez Family Consulting Company, Iowa City, IA 52246, USA; egarzalopez@gmail.com; 2 Department of Molecular Physiology and Biophysics, Vanderbilt University, Nashville, TN 37232, USA; zer.vue@vanderbilt.edu (Z.V.); heather.k.beasley@vanderbilt.edu (H.K.B.); andrea.g.marshall@vanderbilt.edu (A.G.M.); tarabia0702@gmail.com (T.A.R.); vanglarry@gmail.com (L.V.); 3 National Heart, Lung, and Blood Institute, National Institutes of Health, Bethesda, MD 20892, USA; prasannakatti.katti@nih.gov (P.K.); brian.glancy@nih.gov (B.G.); 4 Department of Biology, University of Hawaii at Hilo, Hilo, HI 96720, USA; kneikirk@hawaii.edu (K.N.); mbiete@hawaii.edu (M.B.); 5 Department of Internal Medicine, Carver College of Medicine, University of Iowa, Iowa City, IA 52242, USA; jacob-lam@uiowa.edu (J.L.); margaretmungai24@gmail.com (M.M.); salma-ashshareef@uiowa.edu (S.A.); jennifer-streeter-1@uiowa.edu (J.S.); 6 Department of Biochemistry, Cancer Biology, Neuroscience and Pharmacology, School of Graduate Studies and Research, Meharry Medical College, Nashville, TN 37208, USA; 7 Microscopy and Cell Analysis Core Facility, Mayo Clinic, Rochester, MN 55905, USA; christensen.trace@mayo.edu (T.A.C.); salisbury@mayo.edu (J.L.S.); 8 Department of Biochemistry and Molecular Biology, Mayo Clinic, Rochester, MN 55905, USA; 9 Department of Cell Biology, School of Medicine, University of Pittsburgh, Pittsburgh, PA 52013, USA; smurray@pitt.edu; 10 Central Microscopy Research Facility, University of Iowa, Iowa City, IA 52242, USA; jian-shao@uiowa.edu; 11 Fraternal Order of Eagles Diabetes Research Center, Iowa City, IA 52242, USA

In the original publication [1], the legend of Figure 3 has the number “405” in the last sentence. This should be disregarded.

In Section 7.4, instead of “Other useful metrics are available in Amira that are not used here which include mitochondrial branching index and is calculated by the following equation: *SA*^3^/16π^2^*V*^2^ [23]. Mitochondrial complexity measures the ratio between transverse and longitude tissue surrounding the mitochondria [23]”, it should read “Other useful metrics are available in Amira that are not used here, which include mitochondrial complexity index and is calculated by the following equation: *SA*^3^/16π^2^*V*^2^ [23]. Mitochondrial branching index calculates the relative branching between the transverse and longitudinal mitochondrial orientations [23]”.

In Figure 1A,E and Figure 2A, the x- and y- dimensions currently read 10 nm by 10 nm, these should correctly read 10 µm by 10 µm.

The corrected Figure 1 and Figure 2 appear below:

The authors apologize for any inconvenience caused and state that the scientific conclusions are unaffected. This correction was approved by the Academic Editor. The original publication has also been updated.

## Figures and Tables

**Figure 1:**
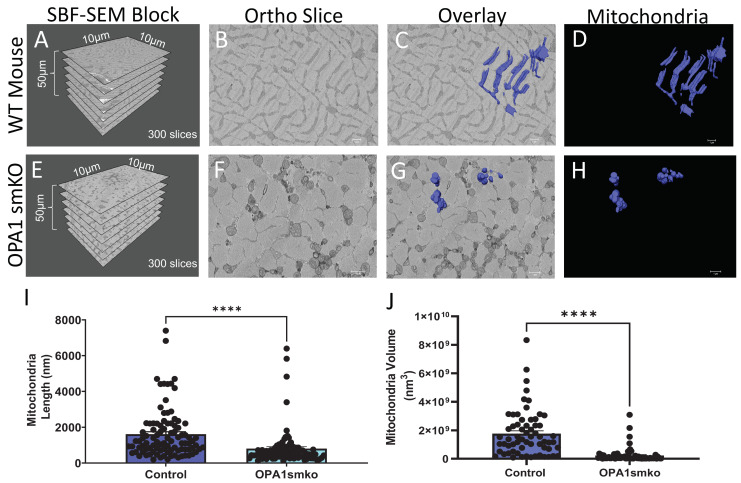
Skeletal muscle specific knockout of *OPA1* (*OPA1* smKO) in mouse leads to changes in mitochondrial morphology in the mouse. The 3D distribution of single continuous and stationary mitochondria (blue), reconstructed from serial block facing-scanning electron microscopy (SBF-SEM) image stacks of gastrocnemius muscle from *OPA1* smKO mouse (**A**–**H**). (**A**) The dimensions of the captured tissue in wild type mouse and (**E**) OPA1 smKO, (**B**,**F**) along with an example ortho slice for each. (**C**) The overlay of the 3D surface rendering of mitochondria in a wild type mouse, on top of a representative ortho slice and (**D**) the 3D surface rendering of mitochondria alone. (**G**) The overlay of the 3D rendering of mitochondria in OPA1 smKO, on top of a representative ortho slice and (**H**) the 3D surface rendering of mitochondria alone. (**I**,**J**) The 3D mitochondrial length and volume decreased (**** *p* < 0.001) upon OPA1 smKO.

**Figure 2:**
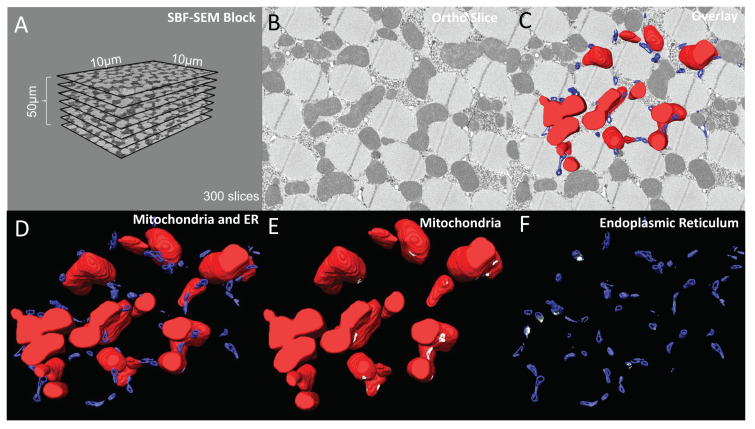
6-panel presentation of 3D reconstruction images and ortho slices from wildtype *Drosophila* flight muscle. This figure is an example of how to present the ortho slices and the 3D reconstruction images. This example shows 3D reconstruction of several organelles in *Drosophila* flight muscle. (**A**) On the left, several representative ortho slices are presented. The dimensions and amounts of ortho slices for data acquisition and conversion to 3D models are shown. (**B**) The raw image of an ortho slice. (**C**–**F**) Mitochondria are colored red, ER are colored blue, and MERCs are colored white. These data are best presented in several ways. (**C**) 3D reconstruction overlaid over the ortho image allows for better visualization of the specific structures in the ortho image that are reconstructed. (**D**) In contrast, the 3D reconstruction not overlaid on the ortho image allows for better visualization of interactions between the 3D structures. (**E**,**F**) Finally, Amira also allows for the graying out of specific structures such that only mitochondria or ER are shown in the 3D reconstruction. This is useful to view otherwise difficult to see areas including MERCs.
