# Supplementary material for: Protocols for Generating Surfaces and Measuring 3D Organelle Morphology Using Amira
Source: Cells. 2021 Dec 27;11(1):65. doi: 10.3390/cells11010065 (PMC8750564; doi:10.3390/cells11010065)
Supplement: Supplementary file 1 [file cells-11-00065-s001.zip › cells-1415914-supplementary mertials.pdf]

**Required Hardware (28):**
1. Wacom Tablet with Stylus[a].<br>a. 16–17 inches are recommended, although smaller tablets may be used.<br>2. Minimum computer specifications:<br>a. Twice as much RAM GB as the data being processed (e.g. 4 GB of data would require 8 GB of RAM).<br>b. 158 GB+ HDD, 50 MB/sec read/write speeds.<br>c. 4 core computer processing unit (CPU) with adequate memory cache for data transfer.<br>d. Graphics Processing Unit (GPU) with OpenGL 2.1, 1 GB GPU memory[b].<br>e. Computer running Microsoft Windows 7/8/10 (64-bit), Linux x86 64, or Mac OS X Sierra (10.12) or later.<br>3. Recommended computer specifications:<br>a. 6 times as much RAM GB as data being processed (e.g. 4 GM of data would require 32 GB of RAM).<br>b. 158 GB+ SSD, 250 MB/sec read/write speeds[c].<br>c. 8 core CPU with large memory cache and fast clock speeds[d].<br>d. GPU with OpenGL 2.1, 16 GB GPU memory[e].<br>e. Computer running Microsoft Windows 10 (64-bit)[f].
- [a]An alternative method that uses the computer mouse rather than the Wacom tablet is available. Other available automation methods are described in the protocol, however, many of these methods have greater noise, thus manual segmentation is recommended. It is possible that alternative tablets may be used, however, this protocol focuses on Wacom tablets due to their easy integration with Amira.<br>- [b]The GPU is the main bottleneck for 3D visualization. Therefore, an integrated GPU is highly recommended against, unless very minimal processing will be done. To maximize 3D rendering, high GPU fill rates and GPU cores should be prioritized when choosing a GPU.<br>- [c]Higher read and write speeds decrease the time spent importing and exporting data. The size of the hard drive does not matter as long as it is adequate to store all the required files and data.<br>- [d]Although a CPU with more than 8 cores may increase the speed of Amira, there is a diminishing return. A large memory cache and clock speed both increase performance, however, the bottleneck typically comes from the GPU, therefore the GPU should be prioritized over the CPU.

- [e]The need for a graphics card depends on the specific project. Memory size is important for volume visualization and for maximizing the resolution of volumetric images. Memory interface and numbers of cores are important for volume rendering and for reducing the time required for rendering. Triangles per second is important for geometric rendering involving surfaces, whereas fill rate is more important for final volume visualization and 3D modeling. Each case will be different, however, for our 3D visualization, we used a powerful graphics card that offered a large memory and a high fill rate.<br>- [f]Due to extensions only available on Windows, including xScreen, xObjectTracking, xWind, xRecipe, and xDigitalVolumeCorrection. Some of these extensions (xScreen, xDigitalVolumeCorrection, and xWind) are offered on Linux Intel64, but none are offered on Mac OS X. Additionally, other features, such as anti-aliasing correction, are absent in Mac OS X, thus Mac OS X is not recommended.

Table S1: Required hardware for 3D reconstruction of organelles and organs using Amira.

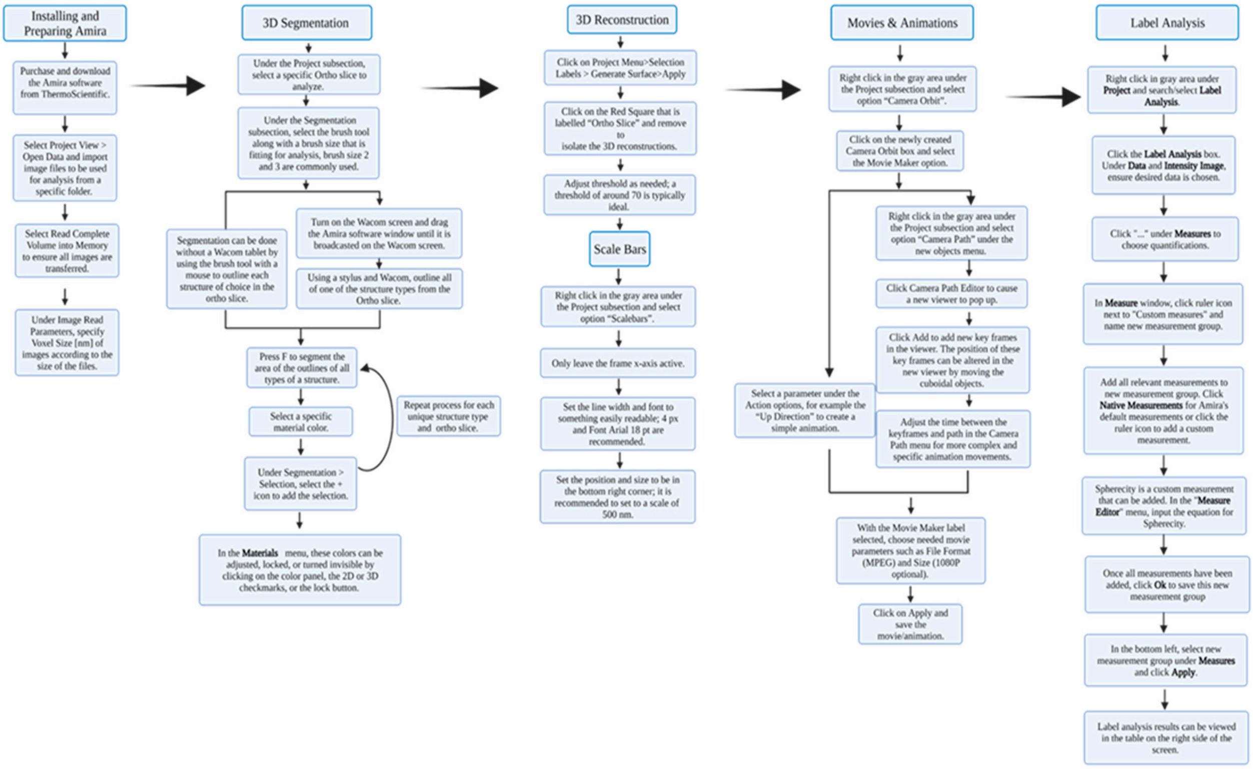

Installing and Preparing Amira
Purchase and download the Amira software from ThermoScientific.
Select Project View > Open Data and import image files to be used for analysis from a specific folder.
Select Read Complete Volume into Memory to ensure all images are transferred.
Under Image Read Parameters, specify Voxel Size [nm] of images according to the size of the files.
3D Segmentation
Under the Project subsection, select a specific Ortho slice to analyze.
Under the Segmentation subsection, select the brush tool along with a brush size that is fitting for analysis, brush size 2 and 3 are commonly used.
Segmentation can be done without a Wacom tablet by using the brush tool with a mouse to outline each structure of choice in the ortho slice.
Turn on the Wacom screen and drag the Amira software window until it is broadcasted on the Wacom screen.
Using a stylus and Wacom, outline all of one of the structure types from the Ortho slice.
Press F to segment the area of the outlines of all types of a structure.
Select a specific material color.
Under Segmentation > Selection, select the + icon to add the selection.
Repeat process for each unique structure type and ortho slice.
In the Materials menu, these colors can be adjusted, locked, or turned invisible by clicking on the color panel, the 2D or 3D checkmarks, or the lock button.
3D Reconstruction
Click on Project Menu>Selection Labels > Generate Surface>Apply
Click on the Red Square that is labelled "Ortho Slice" and remove to isolate the 3D reconstructions.
Adjust threshold as needed; a threshold of around 70 is typically ideal.
Scale Bars
Right click in the gray area under the Project subsection and select option "Scalebars".
Only leave the frame x-axis active.
Set the line width and font to something easily readable; 4 px and Font Arial 18 pt are recommended.
Set the position and size to be in the bottom right corner; it is recommended to set to a scale of 500 nm.
Movies & Animations
Right click in the gray area under the Project subsection and select option "Camera Orbit".
Click on the newly created Camera Orbit box and select the Movie Maker option.
Right click in the gray area under the Project subsection and select option "Camera Path" under the new objects menu.
Click Camera Path Editor to cause a new viewer to pop up.
Click Add to add new key frames in the viewer. The position of these key frames can be altered in the new viewer by moving the cuboidal objects.
Adjust the time between the keyframes and path in the Camera Path menu for more complex and specific animation movements.
Select a parameter under the Action options, for example the "Up Direction" to create a simple animation.
With the Movie Maker label selected, choose needed movie parameters such as File Format (MPEG) and Size (1080P optional).
Click on Apply and save the movie/animation.
Label Analysis
Right click in gray area under Project and search/select Label Analysis.
Click the Label Analysis box. Under Data and Intensity Image, ensure desired data is chosen.
Click "..." under Measures to choose quantifications.
In Measure window, click ruler icon next to "Custom measures" and name new measurement group.
Add all relevant measurements to new measurement group. Click Native Measurements for Amira's default measurements or click the ruler icon to add a custom measurement.
Spherecity is a custom measurement that can be added. In the "Measure Editor" menu, input the equation for Spherecity.
Once all measurements have been added, click Ok to save this new measurement group
In the bottom left, select new measurement group under Measures and click Apply.
Label analysis results can be viewed in the table on the right side of the screen.

Supplemental Figure S1: Printable flowchart of the optimized protocol for organelle quantification using Amira.

Description: Standardized protocol for Amira segmentation, 3D reconstruction, and basic animation creation for organelles. In this process, a Wacom tablet allows for greater autonomy when mapping data elements. Segmenting each ortho slice individually allows for greater control over the process and ensures that high quality and precise 3D images will be generated. In this flowchart, Wacom tablets are used to outline mitochondria and tracheae; however, organelles of any shape or size can be segmented using the proper drawing tools. This flowchart highlights how Amira provides the freedom and flexibility to perform customized tasks and operations. This flowchart may be useful as lab reference material.

Installing and Opening Amira

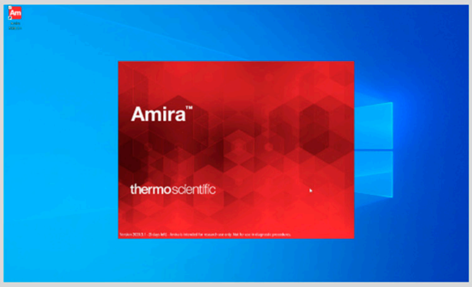

Obtain & launch Amira software from ThermoScientific.

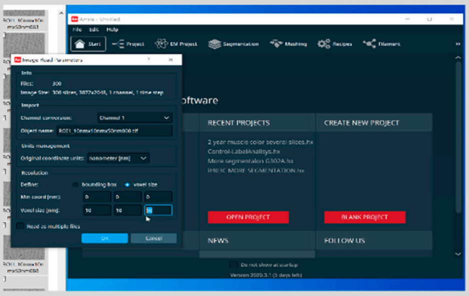

Select Project View > Open Data. Import image files to be used for analysis.

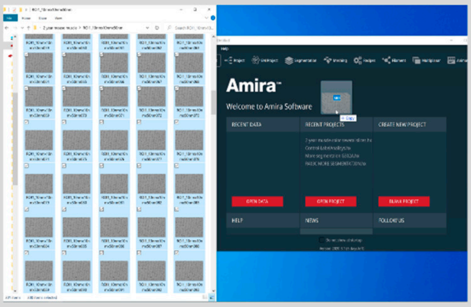

Import images files from a specific folder to Amira

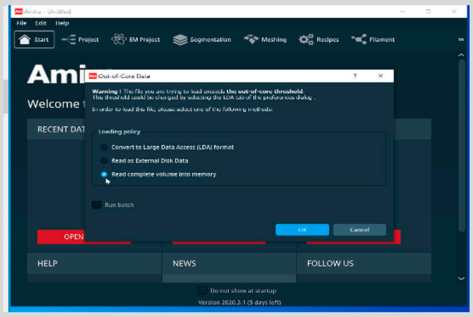

Select Read Complete Volume into Memory to ensure all images are transferred.

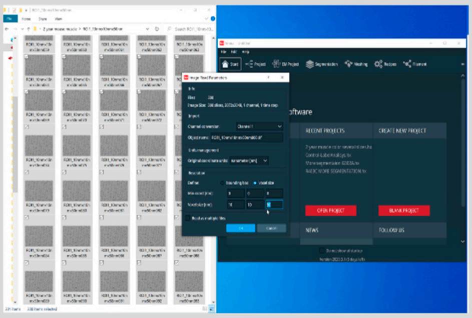

Under Image Read Parameters, specify Voxel Size [nm] of images according to the size of the files.

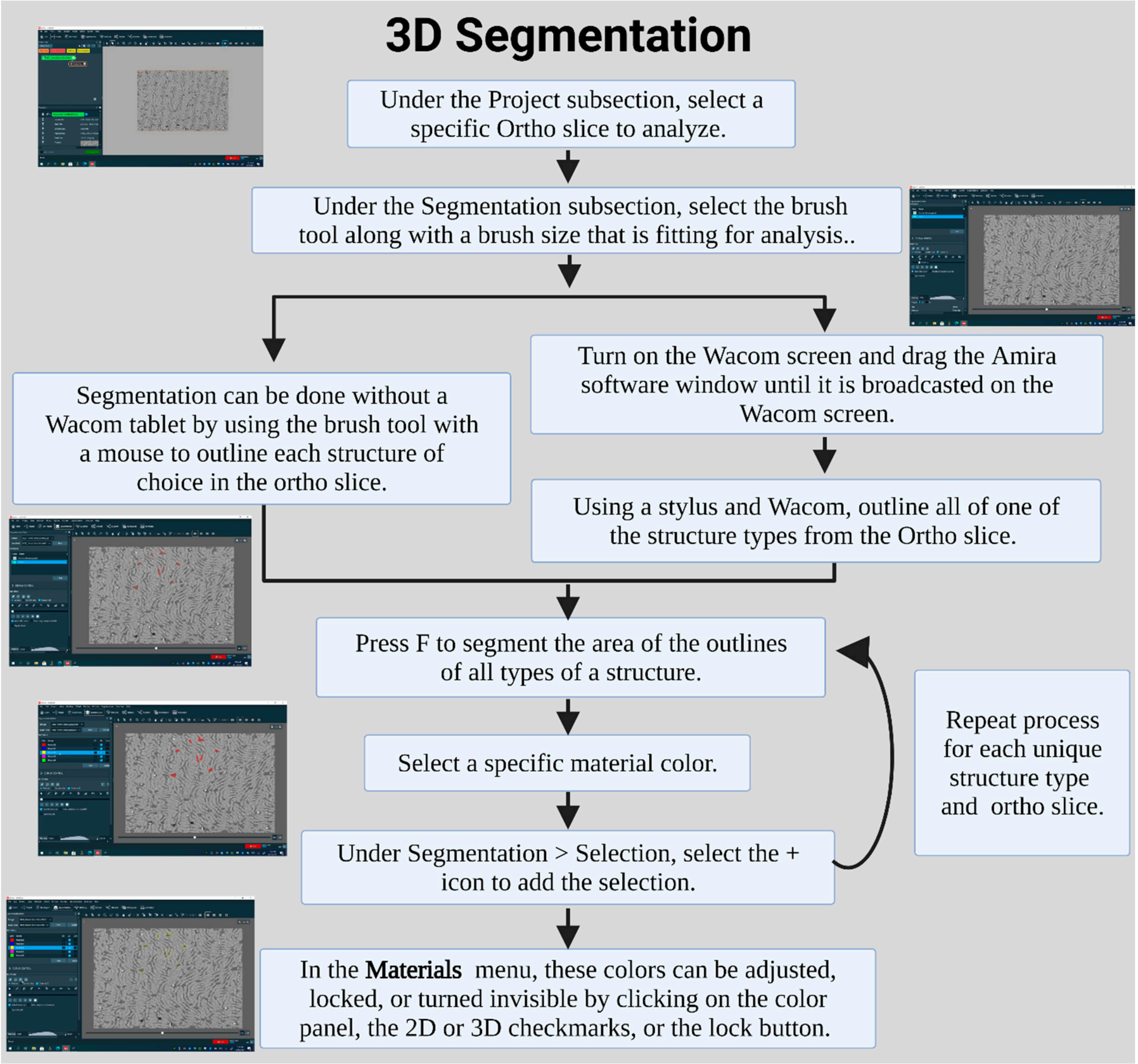
3D Segmentation
Under the Project subsection, select a specific Ortho slice to analyze.
Under the Segmentation subsection, select the brush tool along with a brush size that is fitting for analysis..
Segmentation can be done without a Wacom tablet by using the brush tool with a mouse to outline each structure of choice in the ortho slice.
Turn on the Wacom screen and drag the Amira software window until it is broadcasted on the Wacom screen.
Using a stylus and Wacom, outline all of one of the structure types from the Ortho slice.
Press F to segment the area of the outlines of all types of a structure.
Select a specific material color.
Under Segmentation > Selection, select the + icon to add the selection.
Repeat process for each unique structure type and ortho slice.
In the Materials menu, these colors can be adjusted, locked, or turned invisible by clicking on the color panel, the 2D or 3D checkmarks, or the lock button.

3D Reconstruction

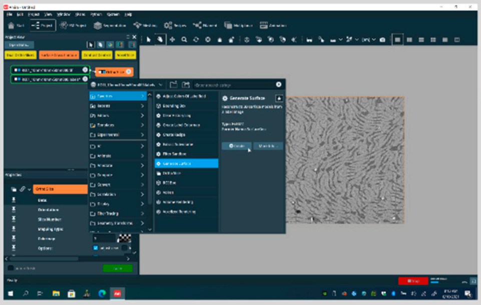

Click on Project view > Selection Labels > Generate Surface > Create

↓

Click on the Orange Square labelled “Ortho Slice” and remove to isolate the 3D reconstructions.

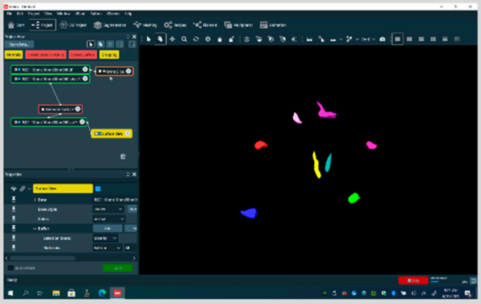

↓

Adjust threshold as needed; 70 is typically ideal.

↓

Scale Bars

↓

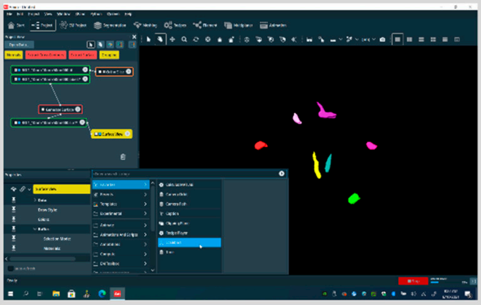

Right click in the gray area under the Project subsection and select option “Scalebars”.

↓

Only leave the frame x-axis active.

↓

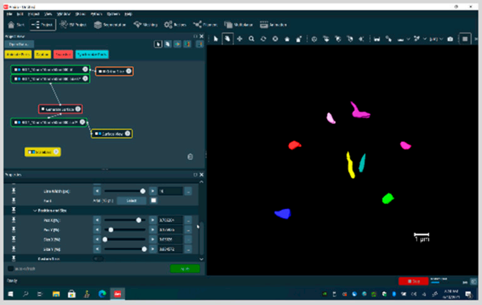

Set the line width and font; 4 px and Font Arial 18 pt are recommended.

↓

Set the position and size in the bottom right corner; scale of 500nm is recommended.

Movies & Animations

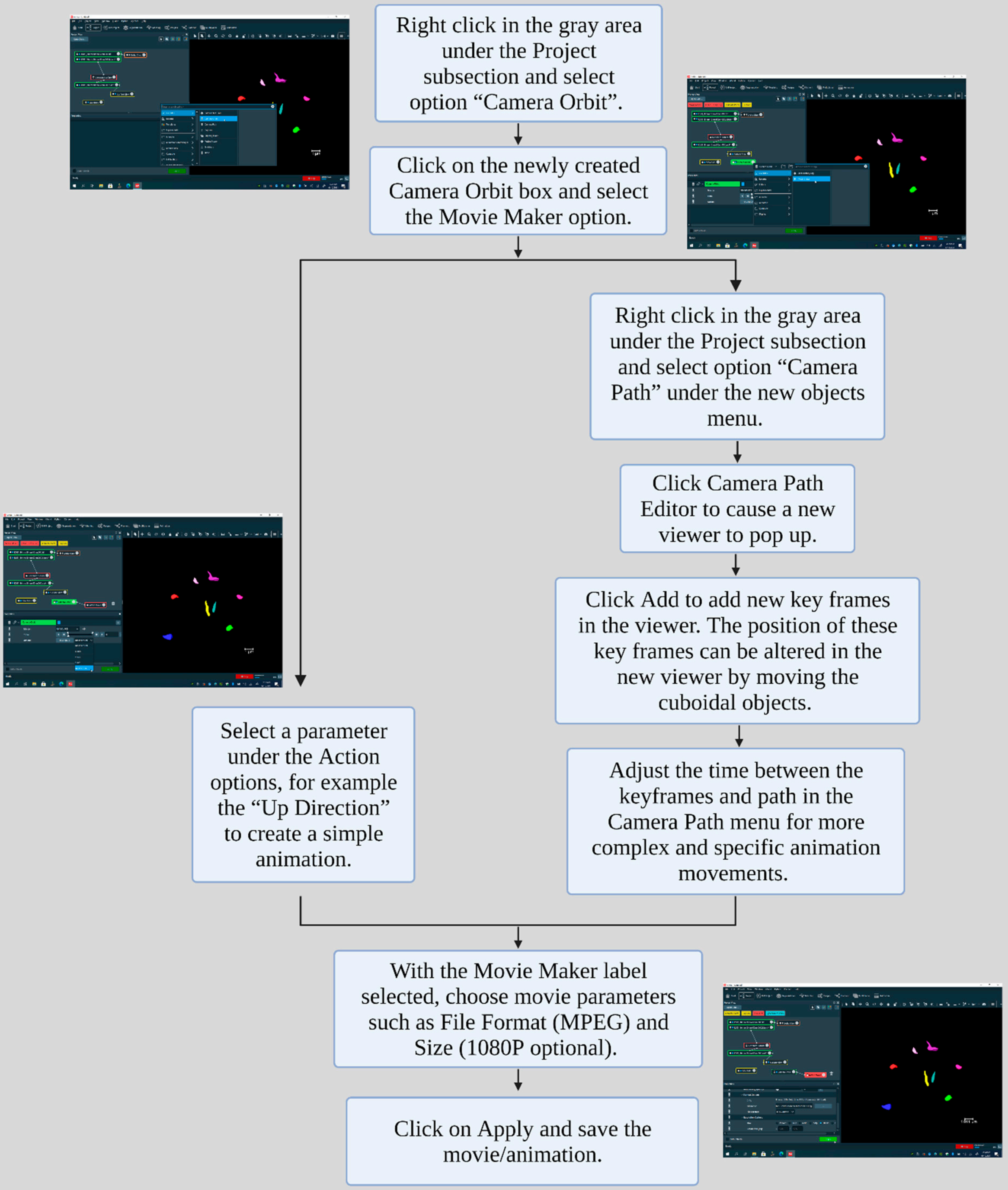

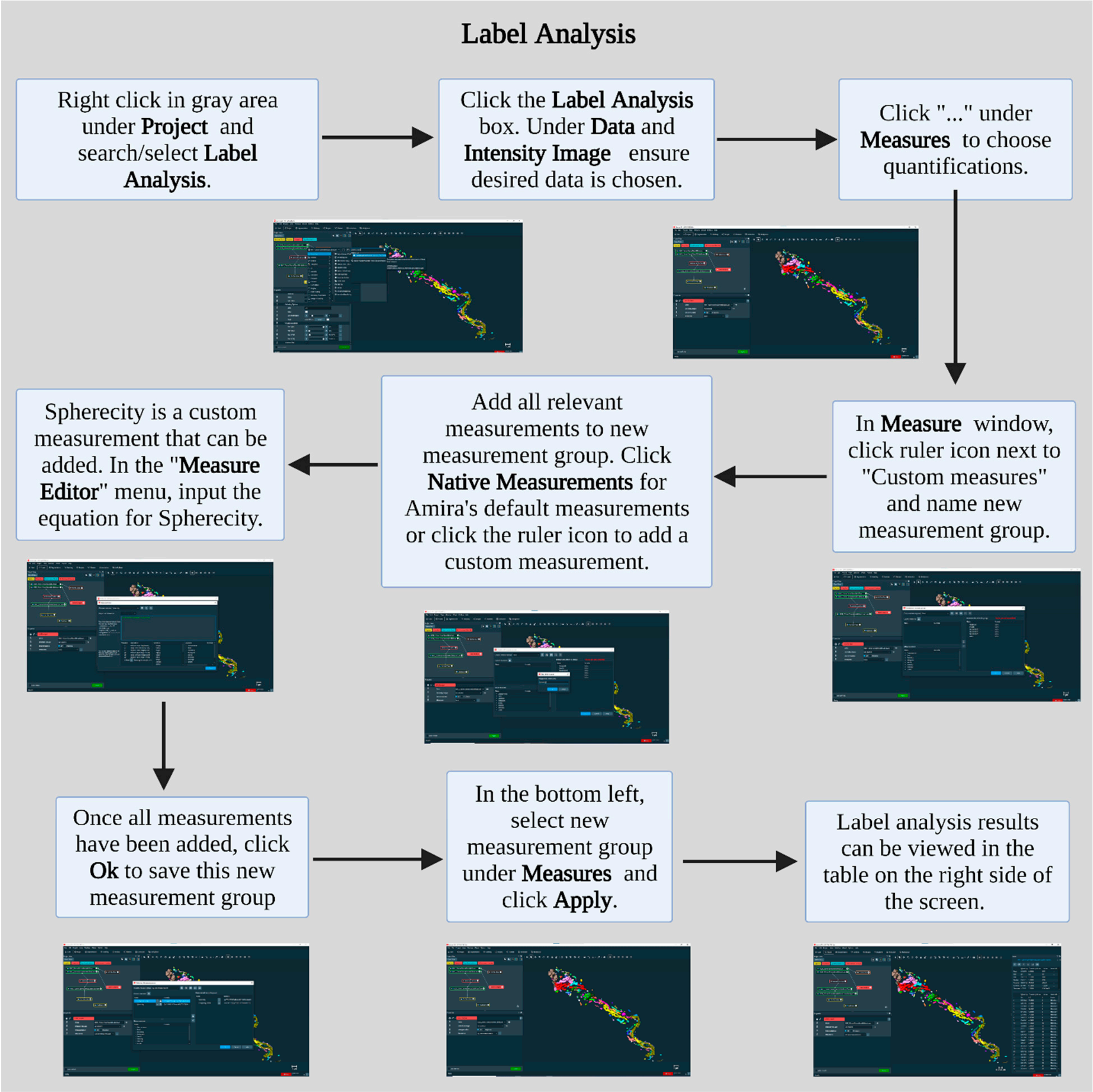

Tables S2–S6: Visualized step-by-step guide for organelle quantification using Amira.

Caption: Amira offers many customizable options, such as animation styles or semi-automated segmentation, that can be adjusted depending on the objectives and desired workflow. Table 1 demonstrates the basic process to open Amira and import photos into Amira. Table 2 demonstrates the process of using a mouse or Wacom table to manually segment each ortho slice, although this may also be performed with other tools as described in the protocol. Table 3 demonstrates the

process of using the segmentation to compile the individual ortho slides into a 3D model of the desired organelles. Table 4 offers a streamlined method for creating videos that can display the 3D models. Methods to create more complex videos are described briefly in the protocol. Table 5 summarizes how to perform quantification using the built in tools on Amira. Although Amira has a high degree of customizability, mastering the software may be difficult. Therefore, this guide provides simple and standardized methods that have many applications and that can be modified or expanded upon as needed.

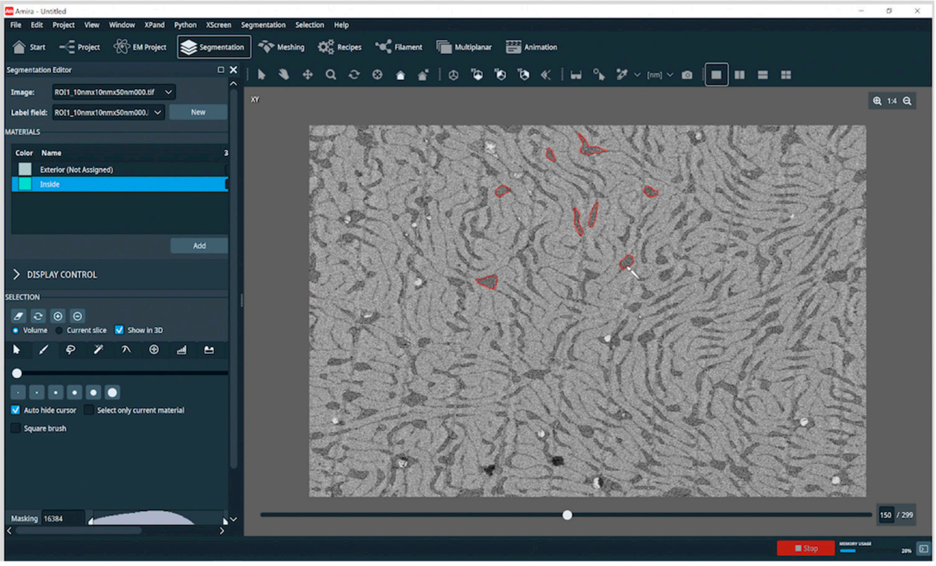

Supplementary Figure S2: Sample of the Amira user interface.

Caption: The segmentation tab on the Amira user interface. To the left is the main control interface that allows for control of the various sections. This methodology requires switching between the project, segmentation, and animation tabs. Under the segmentation section, the bottom left allows for custom control over the brush types and brush sizes. Under the display control subsection, color mapping and zooming may be altered, if relevant. Above the display control section is the materials subsection that allows for various colors, known as materials, to be selected to represent various organelles. In the materials subsection, colors can be modified, names can be changed, and 2D and 3D areas can be selected, adjusted, or locked. Additional materials for separate organelles can be added by selecting the Add button. The main area of the Amira interface contains the image to be analyzed. On this black and white SBF-SEM microscopy image, the highlighted areas show where tracheal segmentation has been performed. Above the image are various tools that allow for control over the function of the mouse, including a pointer, a 3D grabber, a color select tool, and a camera tool. The Project tab (not selected) is important for 3D rendering once segmentation has been completed.

Name of Program	Cost	Coding experience needed	Coding needed	Platform	User Friendliness	Open Source	Other Considerations	Download
3D Slicer	Free	Some	Python	Windows, Linux and macOS	Moderate	Yes	► Offers high flexibility (scalable and widely adaptable) dependent on the user's needs. ► Many options may require strong user knowledge of the software. Not all features are preloaded, so users may need to know what specific features they want[1].	https://download.slicer.org
Amira	Free 21 day trial; Cost is package dependent	No; some	Python with deep learning	Windows, macOS	Moderate	No	► Contains tools for segmentation, alignment, reconstruction, quantification, and video rendering all in one software. ► Very easy to perform basic operations, but options in scripting, algoritms, and extensibility to allow for moroe complex operations[2].	https://www.thermofisher.com/uk/en/home/global/forms/industrial/amira-software-trial.html
Dragonfly Pro	Free 30 day trial; Cost is package dependent	No	N/A	Windows	Moderate	No	► Prebundled with solutions for Deep Learning which offers an easier framework for users to use existing and build convulational new neural networks. ► Deep focus on bringing more accessible form of machine learning to users[3].	https://info.dragonfly-pro.com/home.html
FIJI	Free	No	N/A	Windows, Linux and macOS	High	Yes	► Literature has found that ImageJ/FIJI overestimated the number of certain organelles and clumped together organelles more than other leading softwares. ► Large variety of analysis options, not limited to 3D reconstruction, all with the same overall user interface[4].	https://imagej.net/software/fiji/downloads
Ilastik	Free	Some	Python with deep learning; Fiji and Java	Windows, Linux and macOS	High-to-Moderate	Yes	► Many options for machine learning; however, is limited in other areas and machine learning may still require verification and correction in other softwares such as Amira. ► High compaitability with FIJI, including a plugin and transferable file formats[5].	https://www.ilastik.org/download.html
Imaris	Free 10 non-consecutive day trial; Cost is package dependent	No	N/A	Windows	Moderate	No	► Imaris offers many in-application guides tha can assist in familarizing new users with the software. ► Useful and accurate algorithms implemented in the software that expediates [illegible][4]	https://imaris.oxinst.com/microscopy-imaging-software-free-trial
IMOD	Free	Yes	MATLAB and Python	Windows, Linux and macOS	Moderate-to-Low	Yes	► Ideal for electron tomography reconstruction, with many options for automizing workflows. ► Many options are hidden in the advanced display mode, potentially causing difficulty for new users[6].	https://bio3d.colorado.edu/imod/
MIB	Free	Yes	MATLAB and Python	Windows, Linux and macOS	Moderate-to-Low	Yes	► Ideal for combining manual and automatic workflows, especially for segmentation and alignment of large data sets. ► Limited visualization options; however offers easy export options to IMOD, Amira, and 3D Slicer for better visualization[7].	http://mib.helsinki.fi/tutorials/3D_Modeling.html
Reconstruct	Free	No	N/A	Windows, macOS	High	Yes	► Offers an easy and clear interface that allow for many of the same options (segmentation, basic 3D reconstruction and medium-resolution rendering). ► Compared to other software, more basic options for tracing and editing, alignments, segmentation and 3D surfacing[8].	https://synapseweb.clm.utexas.edu/software-0

Table S7: Major 3D reconstruction software comparison by cost, segmentation options, and other relevant information.

Method Name	Advantages	Disadvantages	Basic Overview of Method
ssTEM	► Best in class x-, y-resolution.<br>► Can be post-stained to increase image contrast.<br>► Modest equipment needs.<br>► Potentially non-destructive[9,10].	► Labor intensive.<br>► Highly dependent on skill of scientist performing it.<br>► Difficult to do in large amount.<br>► Very susceptible to artefacts[9,10].	Conventional TEM thin sections cut by an ultramicrotome with a diamond knife are imaged[9,10].
SBF-SEM	► High z- resolution.<br>► Automized with least reliance on human precision in cutting.<br>► Especially effective for large samples or sets of data[9,11,12].	► Destroys sample.<br>► Cannot do post-staining techniques.<br>► May require secondary machine to remove section debris for complete automation[9,11,12].	An in-chamber ultramicrotome continuously sections and images a sample[9,11,12].
FIB-SEM	► Best z- resolution.<br>► Automized with less reliance on human precision in cutting.<br>► Beam may cut things traditional diamond knives cannot[9,12,13].	► Destroys sample.<br>► Is optimal for smaller volumes.<br>► Requires relatively smooth surface for imaging sample[9,12,13].	An ion beam continuously sections and images a sample[9,12,13].
ATUM-SEM	► Non-destructive.<br>► Mostly automized with less reliance on human precision in cutting.<br>► High x-, y-resolution[12,14].	► Some handling of the sections still required.<br>► More advanced algorithms to properly align and stich orthos in correct orientation for 3D reconstruction needed[12,14].	Serial sections collected by a ultramicrotome with a diamond knife are continuously collected a tape-collecting device[12,14].
Electron Tomography	► Very high overall resolution.<br>► Non-destructive.<br>► Best in class for smaller subcellular structures individually being imaged[9,15].	► Small volumes and thicknesses required, best suited for fewer than a nanometer subcellular structures.<br>► Potential "wedge effect" in which some of the overall object is missing from reconstruction[9,15].	Many 2D tilt angles are collected through TEM and they are utilized together to compile a 3D model[9,15].
Cryogenic EM	► Similar to electron tomography, but reduced chances of artefacts since samples are preserved near native state.<br>► Rapidly evolving and approaching near-atomic resolutions[15,16].	► Much more complicated, and expensive, method than traditional electron tomography.<br>► Not fit for larger and thicker structures.<br>► Potentially lower contrast than other methods[15,16].	Flash-freezing objects to be imaged before tilt angles, while the object remains frozen, are employed to compile a 3D model[15,16].

Table S8: Basic overview, advantages, and disadvantages of main methods of collecting data for 3D reconstruction.

References:

1. Zhang, X.; Zhang, K.; Pan, Q.; Chang, J. Three-Dimensional Reconstruction of Medical Images Based on 3D Slicer. *J. Complex. Health Sci.* **2019**, *2*, 1–12, doi:10.21595/chs.2019.20724.
2. Stalling, D.; Westerhoff, M.; Hege, H.-C. Amira: A Highly Interactive System for Visual Data Analysis. *Vis. Handb.* **2005**, *38*, 749–767.
3. Makovetsky, R.; Piche, N.; Marsh, M. Dragonfly as a Platform for Easy Image-Based Deep Learning Applications. *Microsc. Microanal.* **2018**, *24*, 532–533.
4. Gautier, M.K.; Ginsberg, S.D. A Method for Quantification of Vesicular Compartments within Cells Using 3D Reconstructed Confocal Z-Stacks: Comparison of ImageJ and Imaris to Count Early Endosomes within Basal Forebrain Cholinergic Neurons. *J. Neurosci. Methods* **2021**, *350*, 109038, doi:10.1016/j.jneumeth.2020.109038.
5. Berg, S.; Kutra, D.; Kroeger, T.; Straehle, C.N.; Kausler, B.X.; Haubold, C.; Schiegg, M.; Ales, J.; Beier, T.; Rudy, M. Ilastik: Interactive Machine Learning for (Bio) Image Analysis. *Nat. Methods* **2019**, *16*, 1226–1232.
6. Mastronarde, D.N.; Held, S.R. Automated Tilt Series Alignment and Tomographic Reconstruction in IMOD. *J. Struct. Biol.* **2017**, *197*, 102–113, doi:10.1016/j.jsb.2016.07.011.
7. Belevich, I.; Joensuu, M.; Kumar, D.; Vihinen, H.; Jokitalo, E. Microscopy Image Browser: A Platform for Segmentation and Analysis of Multidimensional Datasets. *PLoS Biol.* **2016**, *14*, e1002340.
8. Fiala, J.C. Reconstruct: A Free Editor for Serial Section Microscopy. *J. Microsc.* **2005**, *218*, 52–61.
9. Schneider, J.P.; Hegermann, J.; Wrede, C. Volume Electron Microscopy: Analyzing the Lung. *Histochem. Cell Biol.* **2021**, *155*, 241–260.
10. Harris, K.M.; Perry, E.; Bourne, J.; Feinberg, M.; Ostroff, L.; Hurlburt, J. Uniform Serial Sectioning for Transmission Electron Microscopy. *J. Neurosci.* **2006**, *26*, 12101–12103.
11. Mukherjee, K.; Clark, H.R.; Chavan, V.; Benson, E.K.; Kidd, G.J.; Srivastava, S. Analysis of Brain Mitochondria Using Serial Block-Face Scanning Electron Microscopy. *J. Vis. Exp. JoVE* **2016**, 54214, doi:10.3791/54214.
12. Lidke, D.S.; Lidke, K.A. Advances in High-Resolution Imaging–Techniques for Three-Dimensional Imaging of Cellular Structures. *J. Cell Sci.* **2012**, *125*, 2571–2580.
13. *Transmission Electron Microscopy* vs. *Scanning Electron Microscopy*; ThermoScientific:.
14. Baena, V.; Schalek, R.L.; Lichtman, J.W.; Terasaki, M. Serial-Section Electron Microscopy Using Automated Tape-Collecting Ultramicrotome (ATUM). *Methods Cell Biol.* **2019**, *152*, 41–67.
15. Ercius, P.; Alaidi, O.; Rames, M.J.; Ren, G. Electron Tomography: A Three-Dimensional Analytic Tool for Hard and Soft Materials Research. *Adv. Mater.* **2015**, *27*, 5638–5663, doi:10.1002/adma.201501015.
16. Bai, X.; McMullan, G.; Scheres, S.H.W. How Cryo-EM Is Revolutionizing Structural Biology. *Trends Biochem. Sci.* **2015**, *40*, 49–57, doi:10.1016/j.tibs.2014.10.005.
